# A Firefly-Inspired Method for Protein Structure Prediction in Lattice Models

**DOI:** 10.3390/biom4010056

**Published:** 2014-01-07

**Authors:** Brian Maher, Andreas A. Albrecht, Martin Loomes, Xin-She Yang, Kathleen Steinhöfel

**Affiliations:** 1 Department of Informatics, King's College London, Strand, London WC2R 2LS, UK; E-Mail: Brian.Maher@kcl.ac.uk; 2 School of Science and Technology, Middlesex University, The Burroughs, London, NW4 4BT, UK; E-Mails: A.Albrecht@mdx.ac.uk (A.A.A.); M.Loomes@mdx.ac.uk (M.L.); X.Yang@mdx.ac.uk (X.-S.Y.)

**Keywords:** protein folding, lattice models, H-P and M-J energy functions, Firefly Algorithm

## Abstract

We introduce a Firefly-inspired algorithmic approach for protein structure prediction over two different lattice models in three-dimensional space. In particular, we consider three-dimensional cubic and three-dimensional face-centred-cubic (FCC) lattices. The underlying energy models are the Hydrophobic-Polar (H-P) model, the Miyazawa–Jernigan (M-J) model and a related matrix model. The implementation of our approach is tested on ten H-P benchmark problems of a length of 48 and ten M-J benchmark problems of a length ranging from 48 until 61. The key complexity parameter we investigate is the total number of objective function evaluations required to achieve the optimum energy values for the H-P model or competitive results in comparison to published values for the M-J model. For H-P instances and cubic lattices, where data for comparison are available, we obtain an average speed-up over eight instances of 2.1, leaving out two extreme values (otherwise, 8.8). For six M-J instances, data for comparison are available for cubic lattices and runs with a population size of 100, where, *a priori*, the minimum free energy is a termination criterion. The average speed-up over four instances is 1.2 (leaving out two extreme values, otherwise 1.1), which is achieved for a population size of only eight instances. The present study is a test case with initial results for *ad hoc* parameter settings, with the aim of justifying future research on larger instances within lattice model settings, eventually leading to the ultimate goal of implementations for off-lattice models.

## 1. Introduction

Research on protein structure and folding has a long history, dating back to the seminal work by Pauling and Corey [1]; see also [2,3,4,5]. From a computational point of view, all-atom protein structure prediction is a challenging task. Interestingly, Finkelstein and Badretdinov [6] approximated the worst case folding time of a protein of length *n* as *exp*(*λ* · *n*^2/3^ ± *χ* · *n*^1/2^ /2) *ns*, where *λ* and *χ* are constants close to unity. In terms of algorithmic complexity, this implies a quasi-exponential structure prediction time with an exponent ≈ *n*^2/3^, if the structure prediction follows the folding path and elementary steps reflect structure transitions. Consequently, protein structure prediction has been shown to be NP-hard for various lattice models [7,8].

A common approach to tackle NP-hard problems are population-based methods and search-based heuristics, such as genetic algorithms [9], tabu search [10], Monte Carlo methods [11], simulated annealing [12,13,14] and quantum optimization [15,16]. Furthermore, simplified energy models are used for approximations of tertiary protein structures, with the most popular being the Hydrophobic-Polar (H-P) energy model [17,18,19,20] and the Miyazawa–Jernigan (M-J) energy model [21,22]. In the H-P model, proteins are represented by chains (within a given lattice) whose vertices are marked either as H (hydrophobic) or P (hydrophilic); H nodes are considered to attract each other, while P nodes are neutral. An optimal lattice embedding is one that maximises the number of H-H contacts, where the underlying justification is the assumption that hydrophobic interactions contribute to a significant portion of the total energy function. The M-J energy model is a more realistic model of the free energy of a given conformation, since it takes into account the interactions between specific pairs of amino acids.

In general, lattice models have been shown to be useful for the study of globular proteins [17,19], of the conformational space induced by (simplified) protein sequences [4,20] and for the analysis of a number of other features important in protein structure prediction, such as long-range interactions in proteins [23]. While typical lattice models account only for the backbone representation of protein sequences, recent research aims at incorporating the space required for side chain packing; see the LatFittool in [24]. As pointed out by Moreno-Hernández and Levitt in [20], protein structure predictions carried out in practice may consist of multiple stages that combine computational models of different complexities, where simplified models are often used in the early stages of structure predictions. In the present study, we focus on lattice models as a test case, with future research aiming at the application of the new search methodology to off-lattice models.

For comprehensive information about recent developments regarding lattice-based protein structure prediction, we refer the reader to [25,26]. In the centre of the present paper is the adaptation of a brand new population-based method to protein structure prediction, namely, the Firefly Algorithm (FA) devised by X.S. Yang [27,28]. To the best of our knowledge, FA has been applied before to protein structure prediction only in [29] (the 2D H-P model).

For the H-P model and face-centred-cubic (FCC) lattices, a new tabu search heuristic is applied in [30] to 21 sequences of a length between 90 and 279. The new heuristic is able to improve on previously known minimum energy values in double digit percentages. The details of how to select favourable intermediate conformations in a population-based search are addressed in [31] (the H-P model and FCC lattices). The information can then be utilised for tabu search or related methodologies. Two energy models, namely the H-P model and a contact matrix similar to the M-J model, proposed in [32], are merged in [33,34]. The authors obtain improvements over existing methods in terms of the root mean square deviation (RMSD, the overall distance between the predicted structure and the native folding identified by X-ray crystallography or nuclear magnetic resonance spectroscopy) for a number of sequences out of 12 proteins with a length ranging from 54 to 160.

In [35], the authors devise a set of general guidelines for the application of Ant Colony Optimisationto protein structure prediction in lattice models. The guidelines are derived from computational experiments on 2D and 3D rectangular lattices combined with the H-P model.

In our study, we try to demonstrate the advantage of the Firefly-inspired method in terms of energy function evaluations, calculated over all individual runs of the population-based method. The results encourage us to analyse further larger instances for the H-P model and to adapt the approach to off-lattice models.

## 2. Experimental Section

### 2.1. Lattice Models

In order to simplify the modelling of protein conformations, and to make computational processing of the structure easier, the common practice is to represent the protein on a lattice. In informal terms, a lattice can be defined as any two- or three-dimensional graph. In our case, we will be using three-dimensional graphs, which are infinite in all three directions. A valid conformation on a lattice is a self-avoiding walk of the graph representation of the lattice, of a length equal to the length of the protein sequence being modelled. We will consider two lattice types in our experiments: the three-dimensional cubic lattice and the three-dimensional face-centred-cubic (FCC) lattice.

The formal definition of lattices is adopted from [26,36,37]: A lattice, Λ, is a subset of ℝ*^N^* defined by:

(1) $$\Lambda =\{v=\sum_{i=0}^{N-1}a_{i}u_{i}|a_{i}\in \text{Z}\},$$

where the vectors, **u**_0_, …, **u***_N_*_−1_, form a basis in ℝ*^N^* and can be represented by the basis matrix **B**_Λ_ = (**u**_0_, …, **u***_N_*_−1_).

The three-dimensional cubic lattice is the simplest lattice we consider, where **B**_Λ_ is given by:

(2) $${\text{B}}_{3\text{Dcubic}}=\left(\begin{array}{ccc} 1 & 0 & 0\\ 0 & 1 & 0\\ 0 & 0 & 1 \end{array}\right).$$

Two nodes **v***_i_* = (*x_i_, y_i_, z_i_*),**v***_j_* = (*x_j_,y_j_, z_j_*) ∊ **B**_3Dcubic_ are neighbours, i.e., connected by an edge, if:

(3) $$\left|x_{i}-x_{j}\right|+\left|y_{i}-y_{j}\right|+\left|z_{i}-z_{j}\right|=1.$$

Therefore, each node in the lattice has six neighbouring nodes, e.g., for **v** = (*x, y, z*), the neighbours are (*x* + 1, *y, z*), (*x* − 1, *y, z*), (*x, y* + 1, *z*), (*x, y* − 1, *z*), (*x, y, z* + 1) and (*x, y, z* − 1).

The FCC lattice is more complex and is a three-dimensional lattice formed around triangles. Each lattice node has 12 possible neighbouring nodes, twice that of the three-dimensional cubic lattice. We consider the FCC lattice, since, even though there are less published results for proteins folded over it, it has been shown that the FCC lattice provides good approximations of actual protein structures (see [14] for the relation to Kepler's conjecture). The basis matrix, **B**_Λ_, for the FCC lattice can be defined by:

(4) $${\text{B}}_{\text{FCC}}=\left(\begin{array}{ccc} 1 & \frac{1}{2} & \frac{1}{2}\\ 0 & \frac{\sqrt{3}}{2} & \frac{\sqrt{3}}{6}\\ 0 & 0 & \frac{\sqrt{6}}{3} \end{array}\right),$$

see [26] and also Section 3 in [36]. Two nodes **v***_i_* = (*x_i_, y_i_, z_i_*), **v***_j_* = (*x_j_, y_j_, z_j_*) ∊ **B**_FCC_ are neighbours according to:

(5) $$(|x_{i}-x_{j}|\leq 1)\&(|y_{i}-y_{j}|\leq 1)\&(|z_{i}-z_{j}|\leq 1)\&(|x_{i}-x_{j}|+|y_{i}-y_{j}|+|z_{i}-z_{j}|=2).$$

For both of these lattice models, a contact between two amino acids is defined as any two amino acids on lattice nodes (*x_i_, y_i_, z_i_*) and (*x_j_, y_j_, z_j_*) that meet their respective neighbouring properties, defined above (Equations (3) & (5)), and that have the extra requirement that the two nodes do not hold *P_n_* and *P_n_*_+1_, where *P_n_* represents the *n*-th amino acid in the protein sequence, *P*.

In our population-based model, we consider a separate infinite lattice for each conformation in the population. Conformations are represented as vectors, and as such, we can measure the distance between them over multiple lattices. Using separate lattices for each conformation prevents a move from not being made due to a node already containing an amino acid from another conformation.

All conformations in the population can move independently, which results in an algorithm that, to a certain degree, can be processed in parallel. For a given number of intermediate steps, *T*, the conformational moves from *S*_0_ to *S_T_* can be processed in parallel, for each conformation individually. It is only after each *T* step that we need all conformational moves to be completed, so that the current best result can be established to become part of the Firefly objective function.

### 2.2. Energy Functions

In a further simplification used to aid computational modelling, bioinformaticians consider simplified energy functions to determine the free energy of any valid conformation. We will consider two energy functions, the simplest, Hydrophobic-Polar (H-P) model proposed by Dill [18], and the Miyazawa–Jernigan (M–J) [21,22] energy model.

The H-P energy model is possibly the simplest energy model in general use. It considers each amino acid to be either hydrophobic or polar. The free energy is calculated as the negative sum of the number of non-sequential H-H bonds in the conformation. Due to its simplicity, the H-P model makes calculating the free energy of a conformation fast, but it does not take into account the interactions between specific pairs of amino acids.

In more detail, let *ᾶ_S_* denote a lattice embedding of the protein sequence, *S*. The embedding is *valid*, if *ᾶ_S_* lies along a non-self-intersecting path within the lattice in such a way that adjacent vertices of the chain, *S*, occupy adjacent locations. We define the set of conformations by:

(6) $${\text{F}}_{S}:=\{{\tilde{\alpha }}_{S}|{\tilde{\alpha }}_{S}\text{is a valid lattice embedding for}S\}.$$

Let *HH_c_* denote the function that counts the number of neighbouring hydrophobic amino acids in *ᾶ_S_* that are not neighbours in *S*, but are neighbours of a distance of one within the lattice. The objective function is then defined by:

(7) $$\text{E}({\tilde{\alpha }}_{S}):=-HH_{c}({\tilde{\alpha }}_{S}).$$

The energy function corresponds to the notation *HP100* introduced in [20], which dates back to the energy function defined in [18]. Apart from *HP100*, Moreno-Hernández and Levitt [20] also consider *HP211*, where H-H contacts are weighted with −2, and H-P, as well as P-P contacts are weighted with −1.

The M-J energy model provides a more realistic model of the free energy of a given conformation, since it takes into account the interactions between specific pairs of amino acids. It is represented as a two-dimensional matrix of contact energies between pairs of amino acids; in our experiments, we will use the contact energies matrix presented in [21,22]. Whilst the M-J model is much more biologically realistic, it is more complicated to both implement and compute.

In the original paper [21], where the energy function is introduced, there are two different interaction matrices. The first matrix, often referred to as MJain the literature, stands for the actual energy value of each bond, while the second matrix, MJb, stands for the pairwise contributions to the total free energy related to the fact that two amino acids are forced to expel a solvent molecule and form a contact. In our study, we use the second matrix. The choice is driven by the fact that benchmarks with a provably optimal energy value in the cubic lattice are available for this matrix, MJb [38].

The total free energy under the M-J pairwise interactions is given by Equation (8), where {*r_i_*} is the set of bead coordinates that define *ᾶ_S_*. Let *AT*(*i*) denote the amino acid type of the *i − th* residue of *ᾶ*, and *e_u,υ_* are the entries of the MJb matrix. We have, for the contact function *D*(*r_i_* − *r_j_*) = 1, if beads *i* and *j* form a contact (that is not a covalent linkage) within the lattice, and *D*(*r_i_* − *r_j_*) = 0, otherwise. We then define:

(8) $$\text{E}({\tilde{\alpha }}_{S})=\sum_{i>j}^{N}e_{AT(i),AT(j)}D(r_{i}-r_{j}).$$

In the evaluation of our methods, we will consider different combinations of lattices and energy functions. We will present the following sets of results: The H-P model over the three-dimensional cubic lattice the H-P model over the three-dimensional FCC lattice, the M-J model over the three-dimensional cubic lattice and a modification of the M-J model introduced in [32] over the three-dimensional FCC lattice. We will use the number of energy evaluations as a performance benchmark of our algorithm.

### 2.3. Benchmark Sequences

#### 2.3.1. H-P Benchmarks

The H-P benchmark problems listed in Table 1 are from [39,40] and represent standard structures in this area of research. The benchmark problems have been studied before by the authors [13,14]; see also [41,42].

**Table 1:** Hydrophobic-Polar (H-P) model benchmark problems (length of 48) from [39,40]. E_bench_ is the benchmark minimum on the 3D cubic lattice and E_nat_ the corresponding value for the face-centred-cubic (FCC) lattice.

ID	structure	E_bench_	E_nat_
HP1	hphhpphhhhphhhpphhpphphhhphphhpphhppphpppppppphh	-32	-69
HP2	hhhhphhphhhhhpphpphhpphpppppphpphppphpphhpphhhph	-34	-69
HP3	phphhphhhhhhpphphpphphhphphppphpphhpphhpphphpphp	-34	-72
HP4	phphhpphphhhpphhphhppphhhhhpphphhphphpppphpphphp	-33	-71
HP5	pphppphphhhhpphhhhphhphhhpphphphpphpppppphhphhph	-32	-70
HP6	hhhppphhphphhphhphhphppppppphphpphppphpphhhhhhph	-32	-70
HP7	phpppphphhhphphhhhphhphhppphphppphhhpphhpphhppph	-32	-70
HP8	phhphhhphhhhpphhhpppppphphhpphhphppphhphphphhppp	-31	-69
HP9	phphpppphphphpphphhhhhhpphhhphpphphhpphphhhpppph	-34	-71
HP10	phhpppppphhppphhhphpphphhpphpphpphhpphhhhhhhpphh	-33	-68

#### 2.3.2. M-J Benchmarks

The M-J sequences taken from [38] are also of a length of 48; see Table 2.

**Table 2:** Miyazawa–Jernigan (M-J) model benchmark problems (length 48) from [38], with minimum energy results for 3D cubic lattices.

ID	structure	E_nat_
MJ1	frtrplnhdfynykiwepfkpadfpkawdrmldhvwdsmaswghqhcs	-25.85
MJ2	cdlppftyrhhgndfwknyemikhwdlwrdmfrafwsdpvkasphqas	-25.92
MJ3	frtpwvshqfyayklmehfkwgdfcrnmdkwidslpdrwnpaphdhas	-26.09
MJ4	kdkihfrmnygypawdaqsvkdltcprdwhfphmrdpshnwelaffws	-25.87
MJ5	endvtmdmdpspclfrihnlprahsfdrfgwhqfdkyhykwkwawaps	-26.15
MJ6	ehdaqldfdwsrwtwhgrnsyhapamyrwpvhdmdkpnpkfkifflcs	-26.24

Along with the sequences from [38], we also consider four sequences analysed in [37] and [33] for 3D FCC lattices; see Table 3. However, the underlying energy matrix in [33] is taken from [32]. Therefore, we executed computational experiments for 3D FCC lattices on benchmarks from Table 3 for the energy matrix defined in [32].

**Table 3:** M-J Model benchmark problems for 3D FCC lattices from [37].

ID	structure	length
4RXN	mkkytctvcgyiydpedgdpddgvnpgtdfkdipddwvcplcgvgkdefeevee	54
1ENH	rprtafsseqlarlkrefnenrylterrrqqlsselglneaqikiwfqnkraki	54
4PTI	rpdfcleppytgpckariiryfynakaglcqtfvyggcrakrnnfksaedcmrtcgga	58
2IGD	mtpavttyklvingktlkgetttkavdaetaekafkqyandngvdgvwtyddatktftvte	61

### 2.4. Firefly Algorithm

Swarm intelligence (SI) and bio-inspired computation have received great interests and attention in the literature. SI-based algorithms, such as particle swarm optimization and the Firefly Algorithm, have simplicity, flexibility and lower time complexity, and thus, they can have many advantages over conventional algorithms [44,45].

#### 2.4.1. Standard Firefly Algorithm

The Firefly Algorithm (FA) was developed by X.S. Yang in 2008 [27,28], which was based on the flashing patterns and behaviour of tropical fireflies. FA is simple, flexible and easy to implement. The basic assumptions in the Firefly Algorithm are:

- All fireflies are unisex, so that one firefly will be attracted to other fireflies regardless of their sex;
- Attractiveness is proportional to the their brightness; thus for any two flashing fireflies, the less bright one will move towards the brighter one. The attractiveness is proportional to the brightness, and they both decrease as their distance increases. If there is no brighter one than a particular firefly, it will move randomly by random walks;
- The brightness of a firefly is affected or determined by the landscape of the objective function.

As a firefly's attractiveness is proportional to the light intensity seen by adjacent fireflies, we can now define the attractiveness, *β*, of a firefly by:

(9) $$\beta =\beta_{0}e^{-\gamma r^{2}},$$

where *β*_0_ ∊ (0,1] is the attractiveness at *r* = 0.

The movement of a firefly, *i*, attracted to another more attractive (brighter) firefly, *j*, is determined by:

(10) $$x_{i}^{t+1}=x_{i}^{t}+\beta_{0}e^{-\gamma r_{ij}^{2}}(x_{j}^{t}-x_{i}^{t})+\alpha \epsilon_{i}^{t},$$

where the second term is due to the attraction. In addition, γ > 0 is a scaling factor and should be related to the scales of the problem of interest. The third term is randomization with *α* being the randomization parameter, and $\epsilon_{i}^{t}$ is a vector of random numbers drawn from a Gaussian distribution at time *t*. Obviously, the randomization term, $\epsilon_{i}^{t}$, can also be drawn from other probability distributions, such as Lévy flights [27,28]. A comprehensive review of the Firefly Algorithm and its variants has been carried out by Fister *et al.* [46].

For this algorithm to reach convergence properly, randomness should be gradually reduced, and one way to achieve such randomness reduction is to use:

(11) $$\begin{array}{cc} \alpha =\alpha_{0}\theta^{t}, & \theta \in (0,1) \end{array},$$

where *t* is the index of iterations/generations. Here, *α*_0_ is the initial randomness factor, and we can set *α*_0_ = 1 without losing generality

As for the parameter values, parametric studies [27,46] suggested that *α*_0_ ∊ [0.5,1], *θ* ∊ [0.7,0.975], *n* = 15 to 100, and γ = 0.01 to 10, though *α*_0_ = 1 and γ =1 can work for many applications.

**Figure 1:**
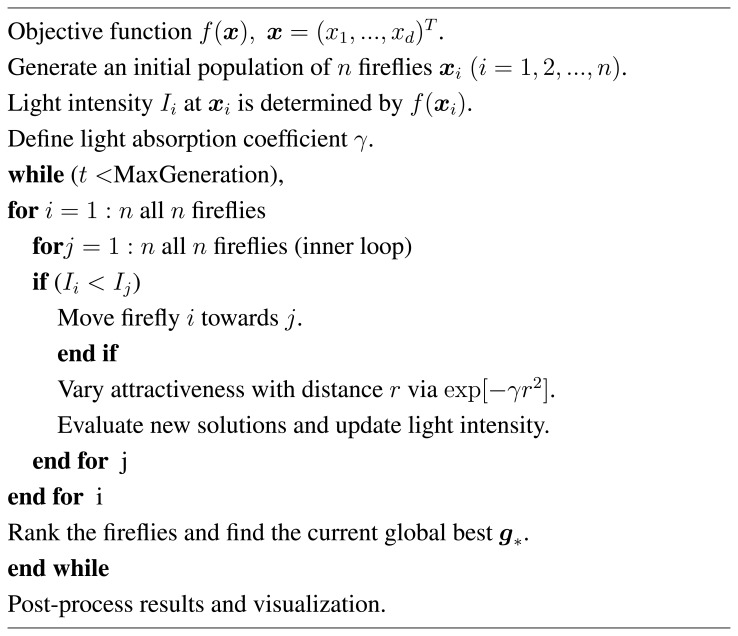
Pseudocode of the Firefly Algorithm (FA).

From the above equation, we can see that mutation can be used for both local and global searches. When $\epsilon_{i}^{t}$ is drawn from a Gaussian distribution and Lévy flights, it produces a mutation on a larger scale. On the other hand, if *α* is chosen to be a very small value, then mutation can be very small and, thus, limited to a subspace. During the updating steps in the two loops in the FA, ranking, as well as selection is used, see Figure 1. A brief analysis of the firefly algorithm can reveal two distinct advantages:

- The automatic subdivision of the whole population into subgroups;
- The natural capability of dealing with multi-modal optimization.

All these advantages make FA very unique and very efficient.

#### 2.4.2. Special Cases of FA

The Firefly Algorithm can have rich characteristics. First, it uses attraction to influence the behavior of the population. As local attraction tends to be stronger than long-distance attraction, the population in FA can automatically subdivide into subgroups, depending on the modality of the problem, which enables FA to deal with multimodal, nonlinear optimization problems naturally.

Furthermore, if we look at the updating Equation (10) more closely, we can see that there are special cases when some parameters are approaching certain limits. Firstly, if γ is very large, then attractiveness or light intensity decreases too quickly; this means that the second term in Equation (10) becomes negligible, leading to the standard simulated annealing (SA) [47]; see Section 2.5.2..

Secondly, if γ is very small (*i.e.*, γ → 0), then the exponential factor $\exp\left[-\gamma r_{ij}^{2}\right]\to 1$. We have:

(12) $$x_{i}^{t+1}=x_{i}^{t}+\beta_{0}(x_{j}^{t}-x_{i}^{t})+\alpha \epsilon_{i}^{t}.$$

Here, if we further set *α* = 0, then the above Equation (12) becomes a variant of a differential evolution [48]. On the other hand, if we replace $x_{j}^{t}$ by the current global best solution, ***g*** *, we have:

(13) $$x_{i}^{t+1}=x_{i}^{t}+\beta_{0}(g*-x_{i}^{t})+\alpha \epsilon_{i}^{t},$$

which is essentially a variant of particle swarm optimization [44] or, more specifically, the accelerated particle swarm optimization (APSO) introduced by Yang *et al.* [49].

Thirdly, if we set *β*_0_ = 0 and let $\epsilon_{i}^{t}$ be related to *x_i_*, then Equation (12) becomes a pitch adjustment variant in harmony search (HS) [50].

Therefore, we can essentially say that DE, APSO, SA and HS are special cases of the Firefly Algorithm. Conversely, FA can be considered as a good combination of all four algorithms (DE, APSO, SA and HS), to a certain extent. Furthermore, FA uses a nonlinear updating equation, which can produce richer behaviour and higher convergence than the linear updating equations used in standard PSO and DE. Consequently, it is no surprise that FA can outperform other algorithms in many applications, such as multimodal optimization, classifications, image processing and feature selection [46].

### 2.5. Firefly-Inspired Protein Structure Prediction

Whilst our approach does not follow the exact ideals of the Firefly methodology, it could be considered a simulated annealing-Firefly hybrid method. We apply the principles of the Firefly optimisation algorithm to a more traditional population-based simulated annealing algorithm, with a primary goal of minimising the number of objective function evaluations (defined as *Oυal_min_* and *Oυal*_avg_ in our results tables) required to reach a solution.

#### 2.5.1. Pull Move Set

The algorithm we have developed uses the pull moves set [10] with the correction suggested by Györffy *et al.* in [51] (see p. 1848, left column) for cubic lattices. We choose the (adjusted) pull move set, because it has been shown to be reversible and complete for cubic lattices [10,51] and FCC lattices [36] (Section 3), meaning that every possible valid conformation is reachable using only the pull move set. We use this pull move set in conjunction with a local search approach, combined with simulated annealing, in order to find an optimum embedding in the lattice. This approach is used together with the Firefly methodology, as shown below, over both the three-dimensional cubic and FCC lattices.

#### 2.5.2. Simulated Annealing

Simulated annealing has long been used [47] as an optimisation method for local search-based problems. We implement simulated annealing to our local search method to avoid becoming trapped in a local minimum during the early stages of computation. Becoming trapped in a local minimum could prevent an optimal embedding from being reached, since the move to an optimal conformation may not be possible if intermediate conformations with a less-optimal energy function value are not considered.

Since the objective of this experiment is to determine the effectiveness of the Firefly optimisation method, we consider only a linear cooling schedule and not a logarithmic cooling schedule.

We use a standard cooling schedule in accordance with:

(14) $$T_{n}=4000\times 0.9999^{n},$$

and a simulated annealing decision based on:

(15) $$e-\frac{\left|\text{E}(S^{\prime })-\text{E}(S)\right|}{T_{n}}$$

This function is used to determine whether a move is to be taken. The value of *n* is not increased for each member of the population, that is, after 10 steps in a population of a size of 5, *n* = 10, not 50. This ensures that population members who are computed sequentially after another have the same probability of accepting a move at any given step.

#### 2.5.3. Population

Our method is a population-based method. We consider a population of a size of *N* = 8 in our experiments, where this number is at the lower end of the settings for *N* discussed in population-based computing (e.g., if values of *N* = 2*^n^* = 4, 8, 16, 32, … are considered). Moreover, due to the multi-threaded nature of our implementation on four CPU cores, the communication overhead is relatively small for *N* = 8.

We create our population by identifying a set of initial conformations. To do this for a population of a size of *N*, we create *N* infinite lattice models. We then make a random self-avoiding walk of each member lattice, of the same length as the protein sequence, attaching the value of each amino acid in the sequence to its respective node. Once we have this random initial conformation, we run a greedy version of the pull move set algorithm on each initial conformation, until we reach a local minimum. This greedy version of the pull move set will only accept moves from the pull move set that result in a lower (better) energy function evaluation.

In traditional simulated annealing combined with population-based methods, the entire population is considered for simulated annealing-based evaluation using the energy function at every step. A crucial feature of our approach is that simulated annealing-based decisions are executed at each step only for the conformation with the lowest value of the energy function (at the last known iteration of *T* steps), whereas the remaining members of the population are evaluated at each step according to their Euclidean distance to the best conformation. The selection of the best conformation is then updated after *T* steps, as described in Section 2.5.4..

#### 2.5.4. Integration with Firefly

We use the Firefly method as a tool for guiding intermediate solutions towards optimum solutions, where the (intermediate) best solution within the population serves as a reference structure for a limited number of *T* steps and changes during this interval according to simulated annealing decisions.

We consider the initial population (*S*_1_ … *S_n_*) and an interval, *T*. At the beginning, pull moves are executed *T* times with the only criterion that the energy value does not increase (greedy descent, potentially to local minima within *T* steps). We then perform an energy evaluation of all conformations in the generated initial population, and save the conformation with the lowest energy value as *S_best_* (random selection, if there are several conformations with the lowest energy value according to Equation (7) or Equation (8)). It is important to note that the member, *S_best_*, of the population remains the same member during simulated annealing-guided conformational changes during each interval of *T* steps. In terms of the standard Firefly Algorithm, *S_best_*, can be considered to be the firefly with the brightest light (and, thus, the light intensity of a population member is inversely proportional to its energy function evaluation; that is, lower energy function evaluations result in brighter firefly lights).

While for each *T*-interval, standard pull moves are executed and evaluated based upon simulated annealing on *S_best_*, for every other conformation in the population (*N* − 1), pull moves are applied with respect to an alternative objective function: In order to mimic the Firefly method, each of these *N* − 1 conformations is evaluated in accordance with its Euclidean distance to *S_best_*, i.e., the second objective function favours changes to the corresponding lattice embedding that move the conformation structurally towards the current *S_best_*.

In formal terms, the structural similarity of [*S_best_, S_i_*] is defined by the Euclidean distance: For the 3D coordinates (*x^t^, y^t^, z^t^*) of the *t* − *th* node (amino acid) at step *n* within the *T*-interval, we set:

(16) $$d_{n}^{t}(i)=\sqrt{{\left(x_{\text{best}}^{t}-x_{i}^{t}\right)}^{2}+{\left(y_{\text{best}}^{t}-y_{i}^{t}\right)}^{2}+{\left(z_{\text{best}}^{t}-z_{i}^{t}\right)}^{2}}.$$

If, now, a pull move affects the positions of *m* nodes (amino acids) from position *j* until position *k* (*m* = *k*−*j* + 1), we select the average value of the distance changes as the structural objective function:

(17) $${\text{sof}}_{n+1}(S_{i})=\frac{1}{m}\sum_{t=j}^{k}|d_{n}^{t}-d_{n+1}^{t}|.$$

The pull move is only accepted for the population member, *S_i_*, if sof*_n_*(*S_i_*) ≥ sof*_n_*_+1_(*S_i_*). Thus, if the total distance increases, but more nodes are affected, the move could still be accepted, which can contribute to the diversity of the population.

Our approach follows the Firefly optimisation principle, since all members of the population with dimmer lights (higher energy function evaluations) move (structurally) to the member with the brightest light (the lowest energy evaluation score).

After *T* steps, we perform an energy function evaluation on all *N* members of the population, and the member of the population that is labelled *S_best_* is updated.

The procedure terminates if there are no pull moves accepted by simulated annealing for the conformation labelled as *S_best_* within the current *T*-interval, and the member of the population labelled as *S_best_* remains the same after this particular interval.

**Figure 2:**
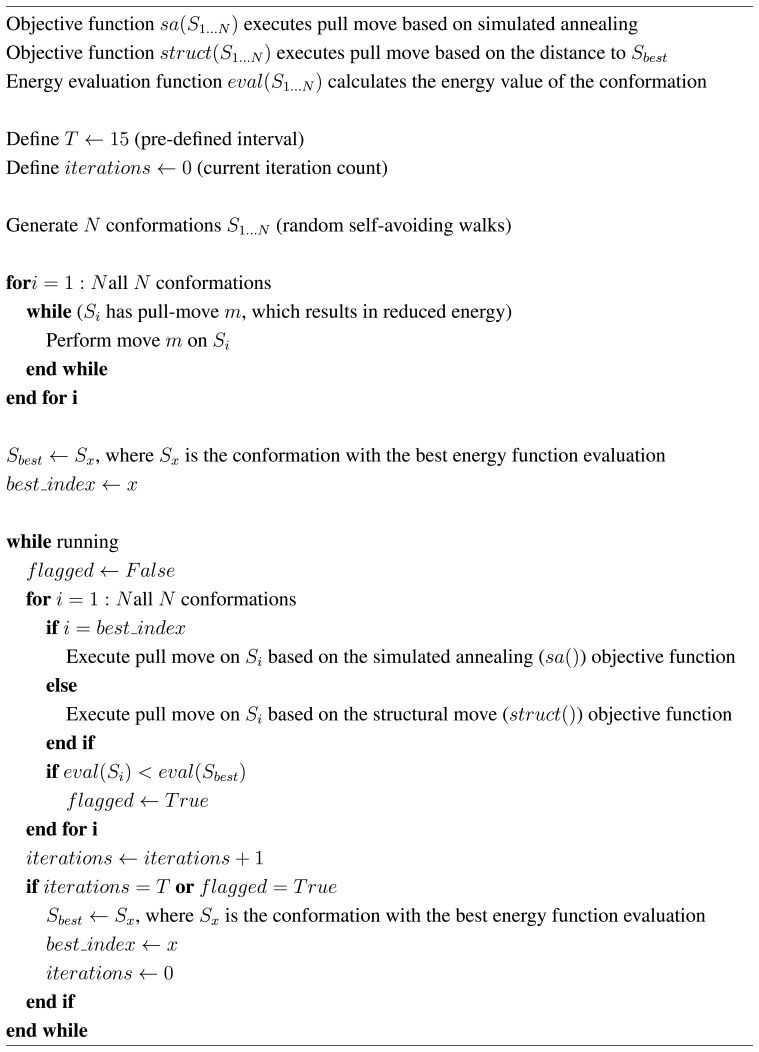
Pseudocode of the integration of the Firefly Algorithm and local search.

As an overview (see the pseudocode in Figure 2), the main steps of the algorithm are:

1. Generate a random initial population of random walks (conformations).
2. Perform pull moves on each conformation greedily until a local minimum is found.
3. Copy the best conformation in the population into *S_best_*. Note its index in *best _index*.
4. Repeat steps 5 through 7.
5. Perform a pull move on conformation *best_index* using the simulated annealing objective function.
6. Perform a pull move on all other conformations using the structural objective function.
7. If we have reached *T* iterations since *S_best_*, we set a new value for *S_best_* and *best_index* by picking randomly from all conformations with the lowest energy function evaluation.

## 3. Results and Discussion

In our experiments, we tested the performance of our algorithm on both the H-P and M-J energy functions on three-dimensional cubic and FCC lattices. We take the minimum and average results and objective function counts from five runs of each combination. All experiments are run with a population size of eight and a default interval of *T* = 15. By selecting such a small value of *T*, we try to avoid that conformations different from *S_best_* do not move for too many steps structurally towards a conformation that is no longer the best with respect to the energy value. Future research will include finding out whether or not one can identify a range of *T* (depending on the sequence length) more appropriate for this particular parameter setting.

The computational experiments were executed on computers with Intel Xeon E5-4603 CPUs and 16 GB of RAM, running Debian 6 x64. The runtimes for the HP1–HP10 sequences ranged from 35 to 142 minutes on the 3D cubic lattice and from 81 to 250 minutes on the FCC lattice. For sequences MJ1-MJ5 over the cubic lattice, runtimes ranging from 89 to 221 minutes were observed. We expect these runtimes to improve with further fine-tuning of the algorithm and parameter settings.

The performance of algorithms is difficult to compare solely based upon runtime values, since the particular runtimes are affected by the underlying hardware and implementation details. Therefore, we provide data about the total number of objective function evaluations, which is denoted by Oval. The notation has been chosen in order to cover both energy function evaluations according to Equation (7) and Equation (8), which are counted for the member of the population labelled as *S_best_* and the number of ‘structure-based’ objective function evaluations according to Equation (17), which are executed for each of the remaining *N* − 1 members of the population. Thus, if *K* = *k* × *T* + *T′* is the number of steps (without the initial stage) until the termination of the procedure, where 1 ≤ *T′* ≤ *T*, then:

(18) $$\text{Oval}=\sum_{i=1}^{N}{\text{Init}}_{i}+N(k\times T+T^{\prime })+(k+1)(N-1).$$

The values of Initi cover the initial stage before the selection of *S_best_*, and (*k* + 1)(*N* − 1) accounts for the selection of *S_best_* at the beginning of each complete *T*-interval and *T′* (we note that the energy value of *S_best_* is known at the beginning of each interval). By selecting such a performance measure, it is also possible to circumvent the runtime impact of how the underlying lattices (which are equal for the same problem setting) are implemented and handled in computational experiments.

In the literature, comparable data with respect to function evaluations are provided in [52] with respect to the H-P model (ten benchmarks from Table 1), and in [41] for the M-J model (six benchmarks from Table 2); in both cases, for the three-dimensional cubic lattice. The function evaluations are counted for the respective energy function, and therefore, we denote by Eval the total number of energy function evaluations. We note that in [52], the value of Eval is equal to the product of the population size (= 20) and the number of ‘generations’. Since the number of ‘generations’ reported in [52] is the average value over 10 independent runs, we use Eval_avg_ for comparison purposes. In [41] (see Tables 9-14 there), 10 independent runs are reported individually with their respective Eval value and minimum energy value. The population size for each of the runs is 100. For comparison purposes, we calculated the average Eval_avg_ value over 10 runs for each of the six M-J instances.

In our computational experiments, we executed five independent runs, and we denote by Oval_avg_ the average number of objective function evaluations according to Equation (18) for each individual benchmark problem. For comparison to [41] and [52], we introduce the parameter:

(19) $$\text{speed}-\text{up}=\frac{{\text{Eval}}_{\text{avg}}}{{\text{Oval}}_{\text{avg}}}.$$

Table 4 displays our results using the H-P model on the three-dimensional cubic lattice. It shows that, over the 10 benchmark problems, our method finds an optimal conformation for every problem. This is comparable to other methods; as *E*_pub_ shows, the method presented in [52] achieved the same performance.

**Table 4:** Results for the H-P model on 3D cubic lattices for benchmarks from Table 1. ${\text{Eval}}_{\text{avg}}^{\text{pub}}$ values are from the results published in [52].

ID	E_bench_	E_pub_	Z_min_	Z_avg_	${\text{Eval}}_{\text{avg}}^{\text{pub}}\times 10^{6}$	Oval_min_ ×10^6^	Oval_avg_ ×10^6^	Oval_max_ ×10^6^	speed-up
HP1	-32	-32	-32	-29.8	7.43	11.28	17.53	24.62	0.4
HP2	-34	-34	-34	-33.4	74.57	7.87	16.02	26.16	4.7
HP3	-34	-34	-34	-32.2	19.19	6.61	12.65	22.97	1.5
HP4	-33	-33	-33	-30.4	12.03	8.95	14.39	23.96	0.8
HP5	-32	-32	-32	-31.2	12.78	4.58	10.33	16.69	1.2
HP6	-32	-32	-32	-30.8	10.63	12.15	17.42	19.08	0.6
HP7	-32	-32	-32	-30.6	917.37	6.35	12.99	15.84	70.6
HP8	-31	-31	-31	-29.0	15.20	6.34	12.98	26.54	1.2
HP9	-34	-34	-34	-32.6	23.95	9.35	15.94	24.62	1.5
HP10	-33	-33	-33	-32.0	42.46	3.88	7.80	17.24	5.4

average speed-up total									8.8

average speed-up, leaving out HP1 and HP7									2.1

Except for HP1, HP4 and HP6, our method improves on the number of energy function evaluations reported in [52], and on eight out of the ten instances, Oval_min_ is smaller than ${\text{Eval}}_{\text{avg}}^{\text{pub}}$. If the worst (HP1) and best (HP7) performances are left out, we achieve an average speed-up of 2.1.

A potential explanation for the relatively large value of ${\text{Eval}}_{\text{avg}}^{\text{pub}}$ for HP7 could be the landscape analysis carried out in [14] for this instance: HP7 exhibits the smallest γ value, which results in a termination criterion larger by about one margin compared to most of the other sequences (see Table 1 in [14]).

Table 5 shows our results for the H-P model over the FCC lattice. This combination shows similar performance to the three-dimensional cubic lattice, in that both our method and the published [53] method found an optimal conformation in 100% of the benchmark problems. We can also see that in 50% of benchmarks (HP2, HP3, HP4, HP5 and HP8), we achieved a better average conformation value than the results published in [53].

**Table 5:** Results for the H-P model on 3D FCC lattices for benchmarks from Table 1. E_pubMin_ and E_pubAvg_ are the published minimum and average results from [53]. Published energy function evaluation counts are not available.

ID	E_nat_	E_pubMin_	E_pubAvg_	Z_min_	Z_avg_	Oval_min_ ×10^6^	Oval_avg_ ×10^6^	Oval_max_ ×10^6^
HP1	-69	-69	-67.37	-69	-66.2	18.98	23.57	29.74
HP2	-69	-69	-66.97	-69	-67.8	25.08	47.97	69.68
HP3	-72	-72	-68.80	-72	-69.8	23.69	32.22	39.77
HP4	-71	-71	-68.10	-71	-69.4	15.89	21.70	27.46
HP5	-70	-70	-67.77	-70	-68.0	18.70	31.70	38.92
HP6	-70	-70	-66.93	-70	-66.2	13.81	23.80	28.37
HP7	-70	-70	-67.57	-70	-65.0	25.59	37.51	42.22
HP8	-69	-69	-66.37	-69	-68.4	21.11	33.52	41.38
HP9	-71	-71	-69.10	-71	-68.8	16.17	23.12	29.40
HP10	-68	-68	-66.47	-68	-65.6	32.14	45.22	53.72

To our knowledge, there are no published energy function evaluation counts for the benchmark problems HP1–HP10 over the 3D FCC lattice. Comparing to our results for the three-dimensional cubic lattice, there is an increase in energy evaluations over all benchmark problems. This is to be expected considering the additional complexity of the 3D FCC lattice.

Table 6 shows our results from five runs for the M-J model on the three-dimensional cubic lattice. In this combination, we achieved an optimal conformation in four out of the six benchmarks. We recall that the ${\text{Eval}}_{\text{avg}}^{\text{pub}}$ values are the average values of energy function evaluation counts over all 10 runs obtained by PLS in [41] for the corresponding benchmark. We note that except for MJ3, not all of the 10 runs reported in [41] reached the optimum value (ranges from six up to nine runs on the remaining instances).

On four of the benchmarks, we achieved the optimum free energy value. With the *ad hoc* parameter settings we used, we missed out by a margin on MJ1 and and MJ4. It should be noted that the speed-up of objective function evaluations in comparison to PLS from [41] is 1.5 and 1.4 on MJ1 and MJ4, respectively. Therefore, we think it is justified to say that, with a slightly higher computational effort, our approach will be able to achieve the optimum free energy values on these two instances. When leaving out MJ3 and MJ5, the average speed-up is 1.2 (otherwise, 1.1). As mentioned before, the population size in [41] is 100, and we presume that with a moderate increase of the population size for the Firefly-inspired algorithms, a stronger speed-up can be achieved. Furthermore, it is important to note that in [41], the runs terminate when a structure with minimum free energy has been found, which is not the case in our computational experiments.

**Table 6:** Results for the M-J Model on 3D cubic lattices for benchmarks from Table 2. E_pub_ are the results published in [41]. ${\text{Eval}}_{\text{avg}}^{\text{pub}}$ are the average energy function evaluation counts for PLSfrom [41].

ID	E_nat_	E_pub_	Z_min_	Z_avg_	${\text{Eval}}_{\text{avg}}^{\text{pub}}\times 10^{6}$	Oval_min_ ×10^6^	Oval_avg_ ×10^6^	Oval_max_ ×10^6^	speed-up
MJ1	-25.85	-25.85	-24.18	-19.58	33.01	15.24	21.56	28.12	1.5
MJ2	-25.92	-25.92	-25.92	-24.16	12.81	17.37	28.33	33.87	0.5
MJ3	-26.09	-26.09	-26.09	-24.92	6.02	14.76	23.79	27.35	0.3
MJ4	-25.87	-25.87	-23.59	-18.30	34.58	18.54	24.59	23.66	1.4
MJ5	-26.15	-26.15	-26.15	-25.01	23.02	7.91	13.20	19.09	1.7
MJ6	-26.24	-26.24	-26.24	-22.73	36.32	13.73	25.96	36.80	1.4

average speed-up total									1.1

average speed-up, leaving out MJ3 and MJ5									1.2

Table 7 shows our results obtained by using the energy function from [32] over the FCC lattice compared to those from [33].

**Table 7:** Results for the energy pairwise interactions energy function from [32] on 3D FCC lattice for the benchmarks from Table 3. *E*_pub_ are the published results from [33].

ID	length	E_pub_	Z_min_	Z_avg_	Oval_min_ ×10^6^	Oval_avg_ ×10^6^	Oval_max_ ×10^6^
4RXN	54	-166.88	-166.21	-158.90	20.05	39.82	52.78
1ENH	54	-153.79	-150.64	-144.12	16.86	35.09	47.52
4PTI	58	-210.29	-213.52	-200.86	23.98	31.88	58.56
2IGD	61	-183.18	-185.15	-179.88	34.71	64.87	111.22

Our results are comparable to [33] on instance 4RXN, worse on instance 1ENHand better on the longer instances, 4PTIand 2IGD. Unfortunately, no data are available in published literature for comparison of the number of energy function evaluations.

## 4. Conclusions

We presented the initial results of protein structure prediction in lattice models by using simplified energy function and a new Firefly-inspired heuristic with *ad hoc* parameter settings. With regard to H-P instances and the minimum number of objective function evaluations, our methods outperform the published results on eight out of ten benchmark problems for cubic lattices. If the average number of objective function evaluations over five runs is compared to the published data over ten runs, the average speed-up is 2.1 over eight instances, where two extreme cases are left out (0.4 and 70.6). For H-P instances on FCC lattices, all ten optimum free energy values are achieved with a relatively small number of objective function evaluations. For the six M-J instances of a length of 48, we obtained on cubic lattices an average speed-up over four instances of 1.2, leaving out two extreme values (otherwise, 1.1). The speed-up is achieved for a population size of only eight instances, whereas the population size producing the data we used for comparison is 100. We presume the speed-up can be improved, if a larger population size is chosen for our Firefly-inspired method. On four selected benchmark problems of a length between 54 and 61, we achieved for the energy matrix proposed in [32] on FCC lattices an improvement of minimum free energy values on two instances in comparison to the results published in [33]. As in the case of H-P instances on FCC lattices, the number of energy function evaluations is comparably small. The results we obtained can be seen as a proof of concept, which encourages us to move forward towards the analysis of longer benchmark problems and off-lattice models.
